# Editorial: Modern machine learning approaches for quantitative inference of gene regulation from genomic and epigenomic features

**DOI:** 10.3389/fpls.2023.1330793

**Published:** 2023-11-24

**Authors:** Michael Banf, Kangmei Zhao, Thomas Hartwig

**Affiliations:** ^1^ Perelyn, Munich, Germany; ^2^ EducatedGuess.ai, Siegen, Germany; ^3^ Department of Plant Biology, Carnegie Institution for Science, Stanford, CA, United States; ^4^ Institute for Molecular Physiology, Cluster of Excellence on Plant Sciences, Heinrich-Heine University, Düsseldorf, Germany

**Keywords:** gene regulation, machine learning, genomic foundation models, epigenomics, transcriptional regulation, gene expression, generative modeling, genomics

Gene regulation is the intricate and highly dynamic process of inducing or inhibiting the expression of individual genes in an organism’s genome. It is orchestrated by a vast array of molecules, including transcription factors and cofactors, chromatin regulators, as well as other epigenetic mechanisms, which allow an organism to control cell growth and differentiation during development. Further, it allows for the adaptation to a variety of environmental stimuli, which is particularly crucial for sessile organisms, such as plants. In turn, disruptions in gene regulation, have been shown to represent a defining feature of a plethora of diseases.

Given its importance in proper cell functioning and adaptability, decoding the architecture of gene regulation has become one of the most pressing tasks in modern (computational) biology. To this end, it has been a long-held ambition to enable quantitative prediction of gene regulation from genomic and epigenomic features alone. Early attempts in the application of modern approaches in machine learning to predict mRNA abundance levels directly from DNA sequence have already yielded promising results (de Boer et al., 2019). More recently, similar to natural language models, propositions for foundation models have been presented that are capable of learning generalizable features from unlabeled genomic datasets. In turn, these genomic foundation models may then be fine-tuned for specialized tasks such as identifying gene regulatory elements (Avsec et al., 2021; Nguyen et al., 2023).

Despite all this amazing progress, a lot of questions remain regarding how individual factors and epigenomic features involved in the gene regulatory apparatus interact within each organism’s vast genomic landscape. To solve real problems, we thereby need to extend our explorations beyond model organisms, particularly within plant bioinformatics. In this sense, this Research Topic presents a variety of approaches, both statistical and learning based, which exemplify the integration of genomic and/or epigenomic datasets to further our understanding of how plants utilize gene regulation to adapt, interact or respond to changes in their environment and, in turn, how this information may benefit increased yields, more effective drug development or more-stress resistant plants.

Ruengsrichaiya et al. propose a machine learning approach to elucidate gene regulatory mechanisms using experimental sequence datasets, including transcription factor binding site as well as DNA binding domain information. Evaluating different machine learning model architectures, the authors demonstrate their approach to accurately predict interactions of plant specific transcription factor binding domains and corresponding binding sites, resulting in the discovery of some yet unknown elements in the cassava sucrose metabolism regulatory pathway.

Smet et al. combine and evaluate the importance of promoter and gene sequences to predict gene expression in rice as a response to heat or drought stress. By comparing genomic feature importance scores for drought and heat stress over time, the authors identify general and stress-specific genomic features selected by their ensemble based random forest model, demonstrating how interpretable machine learning models are able to accurately predict transcriptional responses and enable novel insights in the role of biological sequence features in critical plant adaptation mechanisms.

Qin et al. and Prasad et al. conduct extensive statistical correlation-based analyses to identify putative gene regulatory network modules. Qin et al. focus on a systematic genome-wide analysis of a specific transcription factor family and their involvement in the biosynthesis of flavonoid metabolism within the newly sequenced medicinal plant Spatholobus suberectus. The authors demonstrate a significant connection between members of this transcription factor family and the amounts of biosynthesis of a variety of plant secondary metabolites.

Prasad et al. elucidate processes of gene expression and regulation within fiber development in cotton at different developmental stages, to analyze their role in fiber quality and yield. Using large scale RNA-seq data the authors apply weighted gene co-expression network analysis to identify and cluster regulatory modules. Per regulatory module, promoter analysis is conducted using core promoter genomic sequences of module genes in order to identify module specific cis-regulatory elements as well as corresponding key regulators. As a result, the authors elucidate the transcriptional regulation of fiber development, including cell commitment, initiation, elongation, and secondary cell wall synthesis.

Finally, Su et al. turn towards more recent machine learning approaches based on generative modeling in order to identify tissue-specific highly expressed genes, regulatory modules and specific key transcription factors in soybean. The authors use large-scale soybean transcriptome data, i.e. microarray and RNA-seq, and address the tissue-specific heterogeneity of gene expression, utilizing an adversarial deconfounding autoencoder model to map gene expressions into a latent space and eliminate confounding variables. To extract genes specifically expressed in certain tissues, they propose a lower dimension projection of the gene expression matrix based on a second autoencoder model which is subsequently used to construct tissue-specific gene regulatory networks for a variety of tissues, including leaf, root, seed or nodules.

In conclusion, as guest editors of this Research Topic, we would like to express our sincere gratitude to all the authors for their valuable contributions, to the many reviewers for their work and time as well as to the regular editors and journal personnel for their guidance.

We hope that this Research Topic not only provides insights into and a useful reference about the current state of the art, but also that the reader may draw inspiration from it for his or her own journey in this exciting field of research.

## Author contributions

MB: Writing – original draft, Writing – review & editing. KZ: Writing – review & editing. TH: Writing – review & editing.

## References

[B1] Avsec Ž. Agarwal V. Visentin D. Ledsam J. R. Grabska-Barwinska A. Taylor K. R.. (2021). Effective gene expression prediction from sequence by integrating long-range interactions. Nat. Methods 18, 1196–1203. doi: 10.1038/s41592-021-01252-x 34608324 PMC8490152

[B2] de Boer C. G. Vaishnav E. D. Sadeh R. Abeyta E. L. Friedman N. Regev A. (2019). Deciphering eukaryotic gene-regulatory logic with 100 million random promoters. Nat. Biotechnol. 38, 56–65. doi: 10.1038/s41587-019-0315-8 31792407 PMC6954276

[B3] Nguyen E. Poli M. Faizi M. Thomas A. Birch-Sykes C. Wornow M.. (2023). Hyenadna: Long-range genomic sequence modeling at single nucleotide resolution. arXiv.

